# Lung function imaging methods in Cystic Fibrosis pulmonary disease

**DOI:** 10.1186/s12931-017-0578-x

**Published:** 2017-05-17

**Authors:** Magdalena Kołodziej, Michael J. de Veer, Marian Cholewa, Gary F. Egan, Bruce R. Thompson

**Affiliations:** 1 Monash Biomedical Imaging, Monash University, Melbourne, 3800 Australia; 2 Institute of Nursing and Health Sciences, Medical Faculty, University of Rzeszow, 35-959 Rzeszow, Poland; 3 Department of Biophysics, Faculty of Mathematics and Natural Sciences, University of Rzeszow, 35-959 Rzeszow, Poland; 4 Department of Medicine, Monash University, Melbourne, 3800 Australia; 5 Physiology Service, Allergy Immunology and Respiratory Medicine, Alfred Hospital, Melbourne, 3800 Australia

**Keywords:** Cystic fibrosis, Lung function, Hyperpolarized MRI, Phase contrast

## Abstract

Monitoring of pulmonary physiology is fundamental to the clinical management of patients with Cystic Fibrosis. The current standard clinical practise uses spirometry to assess lung function which delivers a clinically relevant functional readout of total lung function, however does not supply any visible or localised information. High Resolution Computed Tomography (HRCT) is a well-established current ‘gold standard’ method for monitoring lung anatomical changes in Cystic Fibrosis patients. HRCT provides excellent morphological information, however, the X-ray radiation dose can become significant if multiple scans are required to monitor chronic diseases such as cystic fibrosis. X-ray phase-contrast imaging is another emerging X-ray based methodology for Cystic Fibrosis lung assessment which provides dynamic morphological and functional information, albeit with even higher X-ray doses than HRCT. Magnetic Resonance Imaging (MRI) is a non-ionising radiation imaging method that is garnering growing interest among researchers and clinicians working with Cystic Fibrosis patients. Recent advances in MRI have opened up the possibilities to observe lung function in real time to potentially allow sensitive and accurate assessment of disease progression. The use of hyperpolarized gas or non-contrast enhanced MRI can be tailored to clinical needs. While MRI offers significant promise it still suffers from poor spatial resolution and the development of an objective scoring system especially for ventilation assessment.

## Background

Cystic Fibrosis (CF) is an autosomal recessive non-curable condition caused by mutations in the Cystic Fibrosis Transmembrane Conductance Regulator (CFTR) gene, located on human chromosome 7. The CFTR protein is a chloride ion channel and is expressed in many different organs. Deletion of CFTR activity within the lungs leads to dehydration of the airway surface liquid layer, reduction in mucociliary clearance and chronic bronchopulmonary infection [1, 2]. Chronic bronchitis which results from increased pulmonary infections is caused by lower bactericidal activity of the airway surface liquid and reduced ability to clear mucus. Additionally, mucus plugs which are associated with an increase in mucus viscosity are composed of mucins, neutrophils and bacteria and block small and medium airways. In patients with Cystic Fibrosis recurrent bronchopulmonary infections and mucus obstruction lead to chronic airflow obstruction and abnormalities of gas exchange [3].

In Europe Cystic Fibrosis affects 1 to 3500 new-borns [1]. The implementation of a new-born screening test in many countries means patients are often diagnosed shortly after birth. Therefore, they receive effective treatment which results in improved quality and duration of life. The current median life expectancy in United States and Great Britain is 37 years and is increasing due to improved treatments and physiotherapy. It is estimated children born in the 21st century will have a life expectancy of over 50 years [4]. Nevertheless, as the main cause of mortality in CF is lung disease, it is critical that we improve pulmonary diagnosis in patients to better predict adverse events and monitor therapies [5]. Moreover, it is crucial to establish radiation-free techniques which can be easily translated to clinical practice that visualize early stage changes in respiratory function, especially in order to monitor therapeutic interventions and response after lung transplantation.

The overall goal of this review is to understand how to improve respiratory diagnosis and treatment in patients with Cystic Fibrosis in two contexts: (i) clinical respiratory imaging research techniques, and (ii) novel respiratory imaging research applications.

### Methods

A systematic search of PubMed database was performed for years from 2007 to 2016. We looked for records which involved the phrase ‘cystic fibrosis’ and additional phrases:

1. standards of care
2. magnetic resonance imaging (MRI),
3. He-3 MRI,
4. Xe-129 MRI,
5. non-contrast enhanced MRI,
6. phase contrast MRI.

Papers were included if they mentioned respiratory function assessment in quantitative manner or described the standard clinical tests used to assess cystic fibrosis pulmonary disease. Papers were excluded if the information about material, methods and results was insufficient (did not include quantitative data) or was not written in English.

## Clinical respiratory imaging research techniques in Cystic Fibrosis

### Global lung function assessment

There are many different methods to evaluate pulmonary function in patients with CF with the most common method being spirometry. According to the European Cystic Fibrosis Society spirometry should be performed at least once a year in CF patients. From all obtained values, forced expiratory volume in one second (FEV_1_) is the main indicator of disease progression [6]. However, it requires significant respiratory effort and is unsuitable for children under six years of age. Spirometry is a fast and non-invasive way to measure airflow obstruction in medium to large airways, but is less sensitive to small airway function [6, 7].

Another method used for evaluation of small airways is body plethysmography. It is used to measure total lung capacity (TLC) and residual volume (RV) and it can assess volume of trapped air which is increased in CF lung disease [8]. Body plethysmography is very useful to evaluate lung volumes in infants and pre-school children, showing elevated values of residual volume which suggest early gas trapping even in infants with CF and the ability to follow the treatment response [9]. Body plethysmography can also measure Specific Airways Resistance (sRaw), which is a combination of airway resistance and functional residual capacity (FRC) and indicates airflow obstruction. It has been found that sRaw is significantly elevated in CF children compared to healthy subjects and is more sensitive at detecting early lung disease than spirometry [10].

A more sensitive method to detect small changes in the airways is the inert gas washout test. It measures heterogeneity of ventilation during tidal breathing, and is simple to perform especially in young children. The most studied index for the multiple breath washout (MBW) test in patients with CF is the lung clearance index (LCI). LCI is defined as the number of lung turnovers (exhaled volume/functional residual capacity) required to reduce alveolar gas concentration of nitrogen to less than 2% [11]. LCI has been demonstrated to be a sensitive marker of global ventilation heterogeneity in children as well as in adults [12, 13]. Although the method is very promising and simple to perform, it does not give any spatial information and measures total lung function. Non-invasive respiratory imaging in patients with CF offers the promise of measuring localised sites of gas exchange and locating airway obstruction to allow better targeting of physiotherapy treatments and improved treatment monitoring.

### X-Ray based methods

#### Chest X-Ray (CXR)

Chest X-Ray is a fast and widely used method of assessing anatomical changes in the lungs. For patients with Cystic Fibrosis it is recommended to perform a chest X-Ray once a year [7]. However, due to the low spatial resolution, it is only suitable for providing global morphological information such as atelectasis or pleural effusion [14]. Furthermore, it does not give any functional information.

#### High Resolution Computed Tomography (HRCT)

High-Resolution Computed Tomography (HRCT) in patients with Cystic Fibrosis was performed for the first time almost 30 years ago [15]. Since then, HRCT has become the most widely utilised method to assess morphological changes in CF-related lung disease. Due to the high spatial resolution, it has been demonstrated to be more sensitive in detecting pulmonary abnormalities than spirometry [16, 17]. CT is especially helpful in visualizing trapped gas which occurs even in infants not experiencing other pulmonary symptoms [18]. A recent study demonstrated that gas trapping measured by CT correlates well with spirometry [19].

To reduce movement artefact, especially in infants, researchers use controlled ventilation high resolution computed tomography (CV-HRCT). The images are comparable with those from adults undertaking the breath-hold test [20, 21], and the method has been used to detect CF-related lung disease in infants [22, 23]. Recently Sheikh et al., (2015) presented outcomes showing CV-HRCT was sensitive enough to detect improvements in lungs after antibiotic therapy, and thus assessed the effectiveness of the applied treatment [24]. Nevertheless, both HRCT and CXR require significant exposure to X-Rays. Although the radiation induced cancer risk in CF patients undertaking annual CT lung scans is less than 0.5% (the background radiation induced cancer risk is 1.64% [25]), it must be noted that the cumulative radiation dose to multiple CXR may have unknown adverse effects [24, 26].

### Magnetic Resonance Imaging (MRI)

Magnetic Resonance Imaging of the lung is a significant challenge due to a low physical and proton density in the lungs and the air-tissue interfaces that result in weak and/or distorted MR signals. Whilst the field of pulmonary MRI is rapidly developing, intravenous contrast-enhanced lung MRI (IV-CEMRI) with gadolinium based agents still remains the most common pulmonary imaging modality. MRI data sets have spatial resolution which enables analysis of pulmonary perfusion down to the segmental level and provide very high temporal resolution with less than 1.5 s acquisition times. Pulmonary perfusion MRI is suitable to assess bronchoarterial perfusion changes and to localize perfusion defects in Cystic Fibrosis [27].

For paediatric patients it is essential to monitor lungs as early as possible because morphological changes (i.e. mucus plugging and airways inflammation) occur during the first few months of life and are usually not reflected in noticeable functional measures, e.g. FEV_1_ even until 4 years [28]. A significant advantage of MRI is that there is no ionising radiation. This is particularly important for young patients as developing tissues are particularly sensitive to radiation exposure. To determine if MRI is sensitive enough to detect early symptoms of Cystic Fibrosis, Wielpütz et al. (2014) compared structural and perfusion abnormalities in healthy (*n* = 26) and CF (*n* = 50) infants and preschool children. All subjects were examined with a morphological MRI protocol (turbo spin echo /TSE/ sequence in tidal breathing) and an IV-CEMRI perfusion protocol (3D fast low angle shot /FLASH/ sequence). Abnormal lung structure and perfusion defects were clearly visible in MR images in the CF group [29]. A recent comparison of HRCT and IV-CEMRI in CF children showed a strong correlation between CT and MRI and almost 80% inter-observer concordance for bronchiectasis and mucus plugging [30].

For adult CF patients, systematic monitoring of lung changes is crucial. HRCT and CXR deliver a radiation dose which is cumulative over time and becomes more important with an increasing life expectancy of CF patients. Similar to paediatric MRI, a comparison of CT and CXR with IV-CEMRI outcomes in adult CF patients demonstrated approximately 80% concordance for bronchiectasis and mucus plugging [31]. Intravenous contrast enhanced pulmonary MRI has limitations, with the primary issue being insufficient sensitivity due to the low proton density in lung air spaces. Moreover, IV-CEMRI contrast agents can cause adverse allergic reactions and intravenous catheterisation is a moderately invasive protocol. Despite these limitations, IV-CEMRI is a very promising method for determining perfusion and lung imaging in Cystic Fibrosis patients.

## Novel respiratory imaging research applications

### Hyperpolarized gas MRI in Cystic Fibrosis patients

To effectively image ventilation, hyperpolarized gases (typically He-3 or Xe-129) can be administered prior to subsequent magnetic resonance imaging. The hyperpolarized spin state is generated at a very low spin temperature and is in disequilibrium with the temperature of the sample. The most popular method to produce polarized noble gases is via spin exchange optical pumping (SEOP) utilizing vaporized alkali metal [32].

#### Hyperpolarized helium-3 MRI

Imaging of lung ventilation in patients with Cystic Fibrosis using hyperpolarized helium-3 magnetic resonance imaging was first published in 1999 [33]. Following this, hyperpolarized helium-3 MRI (HP ^3^He MRI) has been examined in multiple settings and has shown high sensitivity for the detection of ventilation impairments [34–44]. This typically requires production of hyperpolarized gas on site which is then mixed with nitrogen and administered to patients with a one litre Tedlar Bag. Most MRI protocols require a 9–18 s breath-hold test to obtain ventilation images unaffected by respiratory movement. The most popular and widely described analysis method is the measurement of a ventilation defect (VD), defined as any well-confined lung area, showing no or low signal intensity compared with normal appearing fully ventilated lung areas. The sequential images provide quantitative information about lung function through the regional quantitation of ventilation volume relative to total volume (VV) and ventilation defects per image can then be presented as percentages of total area. The reproducibility of the analysis and technique has been measured at 96% [35, 36]. The HP ^3^He MRI results correlate strongly with spirometry values (*r* = 0.89, *p* < 0.01) and HRCT (*r* = 0.89, *p* < 0.01), and there is also a trend to correlate with chest X-ray (*r* = −0.38, *p* < 0.06) in adults with Cystic Fibrosis. [37]. Moreover, Forced Expired Volume in one second (FEV_1_) values correlate better with HP ^3^He MRI than with CXR measures [38]. The ventilation volume (VV) and ventilation volume percentage (VVP) as measured by HP ^3^He MRI correlate positively while the ventilation defect volume (VDV) and the ventilation defect percentage (VDP) negatively correlate with equivalent spirometry and HRCT results in CF patients [38, 39].

HP ^3^He MRI is useful to assess different treatments and progression of CF disease by measuring ventilation volumes. The effectiveness of an eleven day intervention with intravenous tobramycin, beta-lactam antibiotic, inhalation of 2.5 mg of recombinant human DNase administrated daily, inhalation of 4 mL of 7% hypertonic saline and chest physiotherapy twice a day was monitored by HP ^3^He MRI and showed a 25% increase in VV in treated patients [39]. Improvement in VV was observed despite no changes in FEV_1_ [40, 41] indicating that HP ^3^He MRI may provide greater sensitivity in detecting changes. Unfortunately, there were no noticeable effects of a single bout of chest physical therapy (CPT), however one CPT session may not have been sufficient to visualise effects as effective physiotherapy is an ongoing process and not a single event [41, 42]. A study by Paulin et al. evaluated the progression of CF disease by measuring the VDP in five adults and repeating the measure after four years. There was a 15% increase in the VDP with a near equivalent decrease in the amount of airflow obstruction (FEV_1_/FVC) ratio. Moreover, they observed a strong correlation between HP ^3^He MRI and spirometry outcomes (*r* = 0.98, *p* < 0.001) [43].

**Fig. 1:**
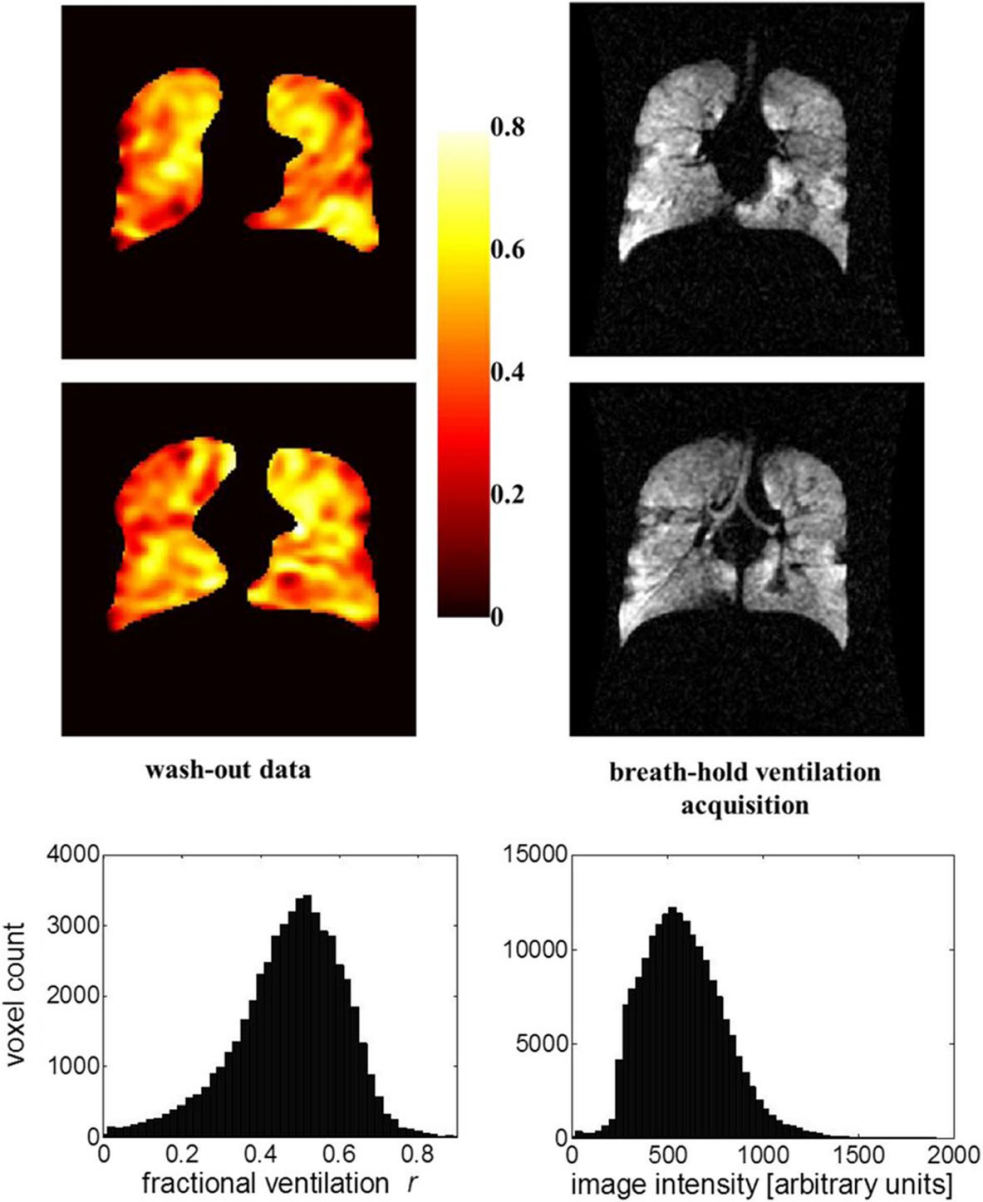
Comparison between HP He MRI breath-hold images and MBW protocol maps in CF patient. Reprinted with permission from Horn FC et al. Journal of Applied Physiology 2014;116:137 [44]

The measurement of VDV is based on counting ‘darker’ and ‘lighter’ areas of HP ^3^He MRI lung images and gives little information about the heterogeneity of ventilation. However, in 2014 Horn and co-workers (Horn et al., 2014) presented a new study protocol based on a combination of hyperpolarized helium-3 MRI and the multiple breath washout (MBW) test. This approach allows the imaging of regional fractional ventilation (RFV) and provides more information about ventilation quality itself, instead of the lung ventilated area only. Nevertheless, the method requires further testing and adjustments and only one CF patient was tested to check the tolerability of the protocol (Fig. 1) [44].

#### Hyperpolarized xenon-129 MRI

^3^He is difficult and expensive to obtain due to the increasing demand for the helium-3 isotope in non-MRI applications and it is typically only sourced from the tritium decay in nuclear warheads [45] making the development of an alternative HP MRI noble gas highly desirable. Measurement of ventilation defects is also possible with polarized xenon-129, although the signal from ^129^Xe is not as strong as ^3^He [45]. The primary issue beyond sensitivity is safety as xenon has anaesthetic properties at higher concentrations. It has been documented that a few inhalation cycles of pure HP ^129^Xe during an imaging session are well tolerated in patients with chronic obstructive pulmonary disease [46, 47]. During imaging ^129^Xe can be diluted with N_2_ if required [47]. Unlike ^3^He, ^129^Xe undergoes a significant chemical shift following dissolution of the gas in the blood further reducing its MRI signal. However this property can be used to measure the dissolved phase, which allows assessment of pulmonary dissolution and perfusion in real time [47, 48].

Diffusion weighted imaging of hyperpolarized gas is possible within the pulmonary system, and is another modality which is very useful for assessing alveolar space [47]. Free diffusion of HP ^129^Xe can be observed in the larger parts of the airway tree. However it is reduced in the small airways, bronchioles, alveolar ducts and alveoli, due to non-Gaussian movement of the gas particles in the lung. Calculation of the apparent diffusion coefficient (ADC) allows the visualisation and quantitation of ^129^ Xe gas diffusion into the tissue. ADC measurements are also possible using ^3^He, however ^129^Xe MRI outcomes correlate better with spirometry values (FEV1/FVC ratio *r* = −0.95, *p* < 0.001) and ^129^Xe has a six-fold smaller self-diffusion coefficient which means smaller field gradients can be used [48]. The unique properties of ^129^Xe allows MR imaging of the gas transition from the alveolar air spaces across the lung wall into red blood cells (RBC). The relative signal intensity from^129^Xe dramatically reduces as ^129^Xe transitions from air to dissolution in interstitial fluid and blood. This causes issues for imaging the transition as the signal is almost immediately overlapped by the diffusion signal and it is difficult to separate the barrier dissolution phase from the RBC dissolution phase due to the small chemical shift difference (19 ppm) and short relaxation time (2 ms) [49].

A new imaging protocol based on an adapted Dixon technique (water/fat separation) called xenon alveolar capillary transfer imaging (XACT) can be used to differentiate barrier and RBCs dissolved phases [49]. A modified Dixon algorithm coupled with a 3D radial pulse sequence during a breath-hold test was used by Kaushik et al. to obtain quantitative images of lung physiological function in patients with idiopathic pulmonary fibrosis (IPF) [50]. Three consecutive images of ventilation, barrier and red blood cell (RBC) ^129^Xe distribution were acquired and a homogenous pattern was found in all three images among healthy volunteers. In IPF patients only ventilation and barrier ^129^Xe images were homogenous and RBC image intensities were lower, mainly in peripheral and basal lung. The calculated gas-transfer defect percentage for IPF patients (22.4% ± 5.4%) was almost two times higher than in the healthy group (11.5% ± 1.3%) [50]. The time taken for magnetization replenishment of ^129^Xe is longer in emphysematous mouse lungs compared to healthy controls. While this method demonstrated that it is feasible to assess ventilation defects, it will be difficult to use in humans due to xenon’s anaesthetic properties and the requirement for constant infusion [51]. A small trial using HP ^129^Xe MRI in Cystic Fibrosis patients (3 adults) demonstrated that 500 ml ^129^Xe + 500 ml 4He, 12–14 s breath-hold, was well tolerated by all participants and showed no significant changes in oxygen saturation and heart rate before, during and after ^129^Xe administration [52].

### Non-contrast enhanced MRI in Cystic Fibrosis

Even very well tolerated contrast agents may cause severe allergic reactions in rare cases, therefore the development of new techniques allowing the measurement of pulmonary perfusion without contrast agents would be extremely useful in these situations [53]. Despite difficulties arising from low proton density, protocols for imaging pulmonary MRI without the use of contrast agents have been possible for over 20 years [54]. Recent advances have improved these protocols and non-contrast-enhanced pulmonary MRI is attracting renewed interest. There are four main MRI techniques for lung imaging without exogenous contrast agent in Cystic Fibrosis.

Arterial Spin Labelling (ASL) is a useful, non-contrast-enhanced MRI technique. It allows assessment of lung perfusion by magnetically labelling water protons within arteries supplying the lungs. Perfusion is measured using a flow-sensitive alternating inversion recovery (FAIR) MRI sequence and then calculated pixel-by-pixel with modified Bloch equations allowing perfusion homogeneity and assessment during tidal, imaging-synchronized breathing. The ASL protocol is well-tolerated by young patients (mean age: 13 ± 5 years) and the outcomes correlate strongly with FEV1 values (*r* = 0.84, *p* < 0.0001) [55]. Mean perfusion values were significantly lower for CF subjects (*p* = 0.02, *p* < 0.05) and the values were even lower for patients older than 15 years (*p* = 0.03, *p* < 0.05) which demonstrates the ability of ASL based techniques to assess disease progression [55].

The ASL technique has also been used to assess pulmonary perfusion after lung transplantation. Lung transplantation is an established treatment for patients with end-stage pulmonary disease. However even though early outcomes show significant benefit the patient is at significant risk of lung rejection known as chronic lung allograft dysfunction (CLAD) [56]. The rejection process is broadly categorized into Bronchiolitis Obliterans Syndrome (BOS) - progressive increase in airflow obstruction and Restricted Allograph Syndrome (RAS) that may be associated with dysfunction in pulmonary vasculature leading to a restrictive ventilator defect [57]. Rejection diagnosis is based on FEV_1_ level and FEV_1_/FVC ratio from spirometry. However, this can be insufficient for patients without clear symptoms of airflow obstruction. The ASL method can be applied to assess pulmonary perfusion in patients after bilateral lung transplant. To evaluate heterogeneity of pulmonary blood flow using MRI, the FAIRER (flow-sensitive alternating inversion recovery with an extra radiofrequency pulse) technique can be used and the comparison of two images enables the calculation of the relative dispersion (RD). Patients with BOS 3 (FEV1 < 50%) have greater perfusion heterogeneity (RD = 1.41) than patients with BOS 0, 1 and 2 (RD_BOS0_ = 1.17, RD_BOS1_&_2_ = 1.06). However patients with the classification of probable BOS (BOS 0p FEV1 81–90% however normal FEV_1_/FVC) have similar perfusion heterogeneity to BOS 3 patients (RD = 1.61). These results suggest this group may be following the RAS pathway where the abnormality is primarily in the pulmonary vasculature with the airways being relatively well preserved [58]. Monitoring of perfusion should be as important as monitoring of airways function since changes in vessels can occur independently from airflow obstruction.

**Fig. 2:**
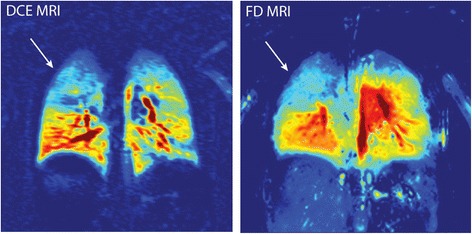
Visual scoring comparison between Fourier Decomposition (FD) MRI and dynamic contrast-enhanced (DCE) MRI (perfusion images from a four-year-old boy). Images present good agreement for localization of disorders and perfusion inhomogeneity. Reprinted with permission form Baumann et al. European Journal of Radiology. 2013;82:2371–2377 [52]

Fourier Decomposition MRI is another technique that has been developed for perfusion evaluation as well as ventilation assessment. It is based on registering the amplitude of local signal strength alterations generated by blood flow during free breathing using short echo-time sequences. Subsequently, collected images are transformed with Fourier analysis to separate the signal from lung parenchyma and blood flow and obtain perfusion and ventilation images. Fourier Decomposition MRI provides results comparable to contrast-enhanced MRI and single photon emission CT (SPECT) [59, 60]. When Fourier Decomposition MRI was compared with contrast enhanced MRI there was consistency between the images (Fig. 2), with similar patterns of perfusion inhomogeneity [59, 60].

Normalized T1 MRI (nT1-MRI) utilizes T1-weighted images, obtained through rapid Look-Locker acquisition to generate a quantitative T1 map of the lung and is able to detect early-stage CF-related lung disease. nT1-MRI is based on the differences in T1-relaxation times between soft tissue (relatively short) and body fluids (relatively long) which gives information about the homogeneity of the dissolution phase within the lungs [61]. A recent study showed that patients with cystic fibrosis had more inhomogeneous nT1 maps than healthy subjects [62]. A significant correlation was found between FEV_1_ and nT1 values (*r* = 0.68, *p* < 0.01) and good agreement was observed between nT1 and ASL to determine regions of oxygen inhomogeneity [61, 62].

Diffusion weighted imaging (DWI) is used extensively to measure water proton diffusion and image inflammation in the brain or abdomen [63, 64]. However, application of DWI for pulmonary imaging can allow for qualitative analysis of the signal intensity of lung defects and can give localised quantitative information about lung inflammation. The technique uses a single-shot echo-planar imaging (EPI) sequence [65]. Recent diffusion-weighted imaging studies in adult CF patients demonstrated strong correlations between MRI and CT (*r* = 0.79, *p* < 0.0001) and FEV_1_ (*r* = 0.688, *p* < 0.0001) values with the highest spatial agreement between mucus plugging and areas with elevated diffusion called hotspots (60%) [65].

The techniques described above offers promise for clinical lung imaging however all require further refinement to improve spatial resolution and disease correlation. The outcomes generally correlate well with standard pulmonary measures and further refinement will improve the spatial resolution, reduce artefacts and allow discrimination of localised lesions within Cystic Fibrosis lungs.

### Phase-contrast X-ray imaging in Cystic Fibrosis

High energy phase-contrast X-ray imaging (PCXI) offers the highest resolution possible for morphological and functional lung imaging. To obtain high-quality images with this technique it is necessary to use X-ray sources with high spatial and temporal coherence. These ultrahigh quality images typically require a synchrotron-based X-ray source [66]. Traditional X-ray imaging uses absorption contrast whereas phase-contrast imaging is based on differences in refractive features of the various materials present within the lung [67].

In patients with CF the dysfunctional CFTR ion channel in the airway cells causes a reduction in the airway surface liquid (ASLiq) volume. The ASLiq layer decrease is associated with a reduction in mucociliary clearance and an exacerbation of disease [66]. Despite the lack of pulmonary symptoms, the mechanisms described occur in CF lungs from the first months of the life, therefore it is critical to apply early treatment and establish imaging techniques that allow for non-invasive ASLiq depth assessment [66]. Currently all PCXI CF lung imaging experiments have been performed in animal models due to the high radiation dose of phase-contrast imaging sources [68–70].

To assess aerosol treatment efficacy, the nasal passages and trachea of anesthetised mice were imaged using a propagation-based phase-contrast X-ray technique before, during and after nebulization with hypertonic saline. Three of seven mice showed significant improvement in ASLiq layer depth (2-5 μm). In contrast to standard X-ray imaging, the PCXI technique can detect borders between different tissues allowing differentiation between the airway lumen and the ASLiq layer [68].

**Fig. 3:**
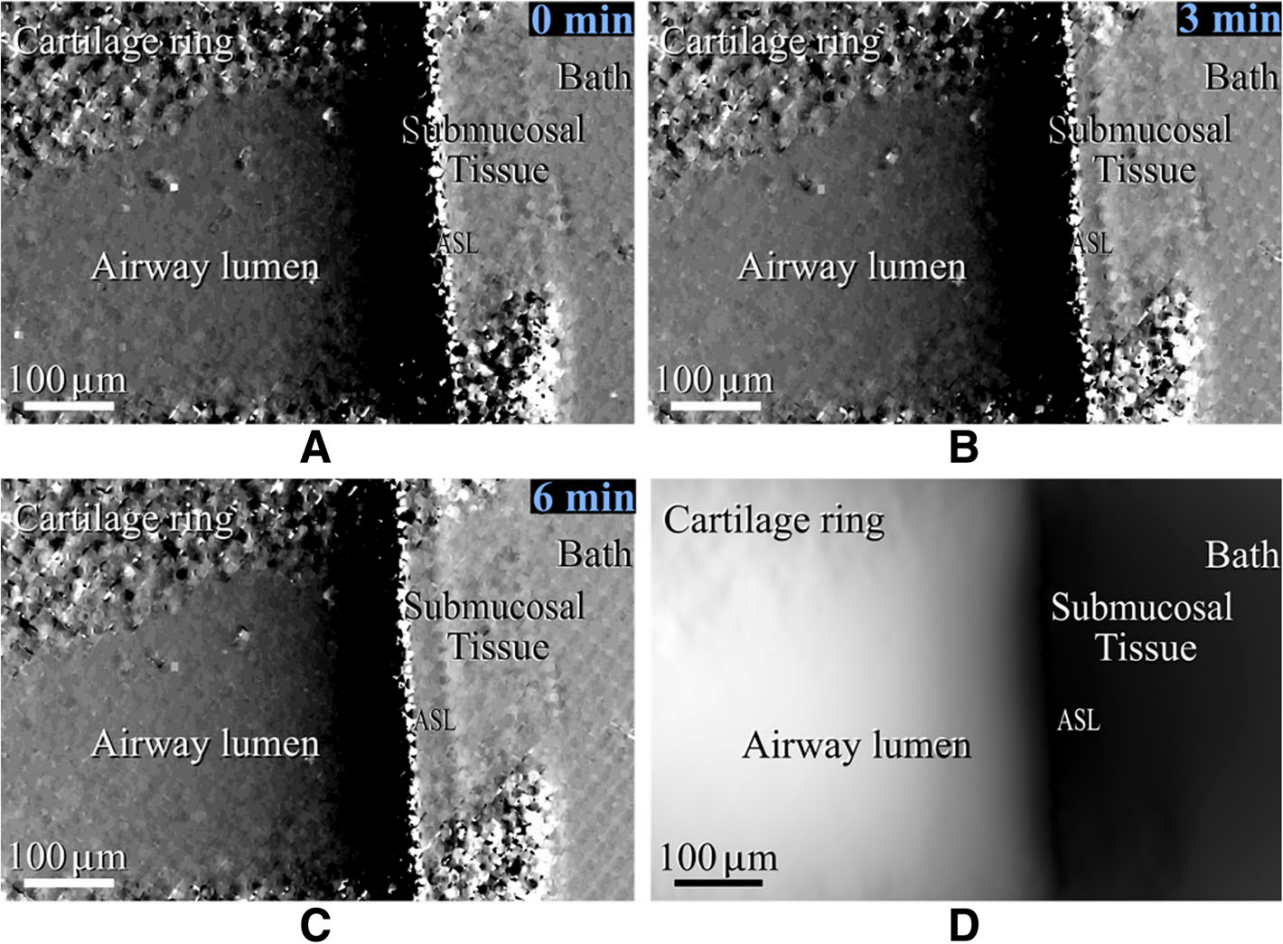
Phase contrast X-Ray based images of the airway surface liquid layer before (**a**), 3 min (**b**) and 6 min (**c**) after hypertonic saline treatment. Projected ASL thickness estimated from image C (**d**). Reprinted with permission from Morgan et al. PLoS ONE 2013;8(1):e55822 [69]

Propagation-based PCXI has a relatively high ability to distinguish ASLiq-to-air differences however it is difficult to distinguish ASLiq and tissue in vivo. Morgan et al. (2013) placed a single absorption grid between the X-ray source and the sample for the ex vivo imaging of murine trachea and were able to determine ASLiq layer volume changes after a hypertonic saline treatment. A statistically significant improvement of ASLiq volume was observed in the treated tracheas (*p* < 0.05) at the micrometre scale (Fig. 3) [69]. Recently the same technique was used for in vivo imaging of CF mice airways and after administrating hypertonic saline statistically significant differences (*p* ≤ 0.0001) were noted from 9, 12 and 15 min acquisitions compared to isotonic saline response [70].

PXCI offers extraordinary detail for the assessment of CF lung disease progression, and while the application of the above techniques in humans is certainly challenging, it offers insight into the mechanisms in pre-clinical animal models and can be used to provide excellent spatial resolution and track the effectiveness of applied therapies.

## Current critical gaps in our understanding

A summary of the techniques can be found in Table 1.

**Table 1:** Summary of respiratory imaging techniques used in patients with Cystic Fibrosis

Method	Technique	Role	Advantages	Disadvantages	Ref
CT	HRCT	**•** Lung anatomy/morphology assessment **•** Disease progression evaluation **•** Treatment response evaluation	**+** quick **+** easy to apply **+** well established in clinical practice **+** high spatial resolution	**-** ionizing radiation method **-** lack of pulmonary function assessment	[16–19]
CT	CV-HRCT	**•** Lung anatomy/morphology assessment in infants **•** Disease progression evaluation **•** Treatment response evaluation	**+** assess lung changes in infants **+** high spatial resolution	**-** ionizing radiation method **-** lack of pulmonary function assessment	[20–24]
MRI	IV CE-MRI	**•** Assessment of pulmonary vascular bed perfusion **•** Lung structure evaluation	**+** Radiation free method **+** pulmonary function assessment (perfusion)	**-** not applicable for all patients (metal parts in body, pacemaker etc.) **-** low spatial resolution due to low proton density of lungs **-** GD contrast can cause allergies	[27, 29–31]
MRI	^3^He MRI	**•** Assessment of ventilation and diffusion functional imaging **•** Disease progression evaluation **•** Treatment response evaluation	**+** radiation free method **+** sensitive track early progression of the disease, early treatment response **+** quantitative information about function (ventilation defect, apparent diffusion coefficient)	**-** not applicable for all patients (metal parts in body, pacemaker etc.) **-** low spatial resolution due to low proton density of lungs **-** currently not applicable to infants due to complicated protocol	[34–44]
MRI	^129^Xe MRI	**•** Assessment of ventilation, diffusion and perfusion possible during one imaging protocol **•** Disease progression evaluation **•** Treatment response evaluation	**+** radiation free method **+** sensitive- track early progression of the disease, early treatment response **+** in-vivo observation of gas dissolution into barrier and RBCs **+** quantitative information about function (ventilation defect, apparent diffusion coefficient, gas-transfer defect)	**-** not applicable for all patients (metal parts in body, pacemaker etc.) **-** low spatial resolution due to low proton density of lungs **-** up to date not applicable to infants due to complicated protocol **-** potentially hazardous due to Xe anaesthetic properties	[46–50, 52]
MRI	Non CE-MRI: ASL	**•** Assessment of perfusion **•** Disease progression evaluation	**+** radiation free method **+** pulmonary function assessment (perfusion) **+** quantitative information (perfusion heterogeneity)	**-** not applicable for all patients (metal parts in body, pacemaker etc.) **-** low spatial resolution due to low proton density of lungs	[55, 58]
MRI	Non CE-MRI: FD MRI	**•** Assessment of ventilation and perfusion **•** Disease progression evaluation	**+** radiation free method **+** pulmonary function assessment (perfusion and ventilation) **+** quantitative information (perfusion homogeneity **+** results comparable to single photon emission CT and CE-MRI but without exogenous contrast	**-** not applicable for all patients (metal parts in body, pacemaker etc.) **-** low spatial resolution due to low proton density of lungs	[60, 61]
MRI	Non CE-MRI: nT1	**•** Assessment of perfusion **•** Disease progression evaluation	**+** radiation free method **+** pulmonary function assessment (perfusion and ventilation) **+** quantitative information (dissolution phase homogeneity)	**-** not applicable for all patients (metal parts in body, pacemaker etc.) **-** low spatial resolution due to low proton density of lungs	[62, 63]
MRI	Non CE-MRI: DWI	**•** Assessment of diffusion changes to localize mucus plugs	**+** radiation free method **+** diffusion changes- localization of areas with elevated diffusion called hotspots	**-** not applicable for all patients (metal parts in body, pacemaker etc.) **-** low spatial resolution due to low proton density of lungs	[65]
Synchrotron based X-Ray	Phase contrast x-ray imaging	**•** Assessment of structural and functional changes in a microscale	**+** very high spatial and temporal resolution of images **+** cimaging function and morphology in microscale	**-** ionizing radiation method- not applicable for humans	[66, 68–70]

There is a strong rationale to monitor the lungs of patients with Cystic Fibrosis at least annually [6]. However, it is important to remember the cumulative radiation dose from methods utilizing X-rays may be harmful. The PCXI method offers the most detailed and informative images, however the currently utilised radiation doses are unacceptably high and the most suitable X-ray source can only be produced using a synchrotron. Reducing the X-ray dose required for effective imaging using PCXI and developing alternate X-ray sources would greatly enhance the applicability of PXCI, however these are difficult goals.

In comparison to X-ray based methodologies MRI has lower spatial resolution for lung imaging. To achieve acceptable signal to noise in an MRI image of the lung it is often essential to use a contrast agent. Lung imaging techniques using non-CE MRI are largely focused on documenting perfusion changes, with poor structural detail available due to the low density of protons and the air-surface interface. To accurately assess ventilation parameters, the use of hyperpolarised gas contrast agent is essential. Although hyperpolarised ^3^He and ^129^Xe are currently used by many researchers, preparing the gas mixture is still challenging since it has to give enough contrast during the examination while being safe for a patient with compromised lungs at the same time. More research investigating the acceptable doses for patients or in pre-clinical models with diseased lungs needs to be performed before effective clinical protocols can be developed. Improvements in the production of hyperpolarized gas would be extremely beneficial, small commercial systems are available however they are not amenable to clinical research and many remain in testing phases and have to be improved before they are ready for more straightforward usage.

Despite the great value of developed methods, the available MRI sequences need significant refinement and testing to minimise the artefacts associated with cardiac and breathing movement. Lung MRI typically involves up to 18 s breath hold which is often a great challenge, especially for young patients. Sequences which allow images to be obtained during free breathing would be of great value for children and patients with advanced pulmonary disease.

Although many MRI techniques have been developed and successfully used for patients with CF, there is still a strong need to perform longitudinal imaging studies to establish clinical protocol. Moreover, the analysis of the images is still problematic, especially for clinical purposes, the use of complicated and time-consuming methods is not practical. Therefore, standardisation of image analysis tools and development of clinically relevant disease markers together with comprehensive clinical tests should be key goals in the future research.

Nevertheless, it is worth noting that MRI allows for dynamic imaging which is critical for CF patients and which provides a unique opportunity to gain insight into lung function in real time.

## Conclusions

The ultimate goal of clinical lung imaging methods in CF is to provide diagnostic indicators and treatment information to clinicians and associated health care workers. Unfortunately, none of the currently available methodologies is simple and detailed enough to fulfil all these objectives. Clearly there is no ‘ideal’ method which allows for the assessment of all CF-associated pulmonary pathology with a single or a few simple modalities. Whilst lung imaging in patients with Cystic Fibrosis is a well-established discipline, there are still many opportunities to improve the imaging methods used. Methods based on ionising radiation should be compared with and if possible replaced by ionising radiation-free methods as any reduction in radiation exposure in CF studies is an important goal to achieve. Magnetic Resonance Imaging requires substantial investment and investigation in CF patients of all ages to improve its clinical relevance. In particular, hyperpolarized ^129^Xe MRI is a poorly developed technique that requires more thorough investigation. Many challenges remain before MRI of the lungs, with or without IV or hyperpolarised gas contrast becomes a routine clinical assessment tool, however MRI appears to have the most potential to become the “gold standard” for regional assessment of cystic fibrosis lung pathology in the future.

## Abbreviations

ADC: Apparent diffusion coefficient
ASL: Arterial Spin Labelling
ASLiq: Airway surface liquid
BOS: Bronchiolitis Obliterans Syndrome
CF: Cystic Fibrosis
CFTR: Cystic Fibrosis Transmembrane Conductance Regulator
CLAD: Chronic lung allograft dysfunction
CPT: Chest physical therapy
CV-HRCT: Controlled ventilation high resolution computed tomography
CXR: Chest X-RAY
DWI: Diffusion weighted imaging
EPI: Echo- planar imaging
FAIR: Flow-sensitive alternating inversion recovery
FAIRER: Flow-sensitive alternating inversion recovery
FEV1: Forced expiratory volume in one second
FLASH: Fast low-angle
FVC: Forced Vital Capacity
HP: Hyperpolarized
HRCT: High Resolution Computed Tomography
IPF: Idiopathic pulmonary fibrosis
IV-CEMRI: Intravenous Contrast-Enhanced Magnetic Resonance Imaging
LCI: Lung clearance index
MBW: Multiple breath washout
MRI: Magnetic Resonance Imaging
nT1-MRI: Normalized T1 Magnetic Resonance Imaging
PCXI: Phase contrast x-ray imaging
RAS: Restricted Allograph Syndrome
RBC: Red blood cells
RD: Relative dispersion
RFV: Regional fractional ventilation
SEOP: Spin exchange optical pumping
SPECT: Single photon emission computed tomography
TSE: Turbo spin echo
VD: Ventilation defect
VDP: Ventilation defect percentage
VDV: Ventilation defect volume
VV: Ventilation volume
VVP: Ventilation volume percentage
XACT: Xenon alveolar capillary transfer
